# Editorial: Opioids and opioid-use disorders

**DOI:** 10.3389/fphar.2023.1227174

**Published:** 2023-06-15

**Authors:** Giordano de Guglielmo, Marsida Kallupi, Andrea Cippitelli, Daniele Caprioli, Kabirullah Lutfy

**Affiliations:** ^1^ Department of Psychiatry, University of California, La Jolla, CA, United States; ^2^ Department of Biomedical Sciences, Charles E. Schmidt College of Medicine, Stiles-Nicholson Brain Institute, Florida Atlantic University, Jupiter, FL, United States; ^3^ Laboratory Affiliated to Institute Pasteur Italia—Fondazione Cenci Bolognetti—Department of Physiology and Pharmacology, Sapienza University of Rome, Rome, Italy; ^4^ Santa Lucia Foundation (IRCCS Fondazione Santa Lucia), Rome, Italy; ^5^ Department of Biotechnology and Pharmaceutical Sciences, College of Pharmacy, Western University of Health Sciences, Pomona, CA, United States

**Keywords:** opioids, opioid use disorder (OUD), novel opioids, overdose, reward, aversion, novel approaches

Opioid analgesics are the mainstay of pain management. However, their acute or chronic use leads to side effects, such as respiratory depression, constipation, tolerance and opioid use disorders, representing major public health and socioeconomic issues.

In this Research Topic, “Opioids and Opioid-Use Disorders: Novel Approaches and Therapeutics,” we have compiled a Research Topic of research articles authored by leading experts in the fields of pharmacology, neuroscience, and addiction research. By harnessing a wide range of methodologies, spanning from *in vivo* animal models to *in vitro* molecular approaches, authors provide scientifically sound evidence in the evaluation of novel opioids and potential pharmacotherapeutic tools for the treatment and prevention of opioid use disorders.

Morphine and other addictive drugs may exert simultaneous reward and aversion and the tag of war between these two effects leads to drug use and misuse or avoidance, this is based on the paradoxical effect hypothesis of abused drugs. Ou et al. measured c-fos, as a marker of neuronal activity, following administration of different doses of morphine in rats using the conditioned taste aversion (CTA) and conditioned place preference (CPP). Then, by using a correlation and network analysis strategy they revealed: 1) in the CTA procedure an association between prelimbic cortex (PrL) and (infralimbic cortex (IL) with NAc core following low doses of morphine. Notably, the authors also observed an association between PrL and IL with NAc shell following higher doses of morphine; 2) in the CPP procedure, an association between PrL and NAc core and shell at low doses of morphine. Again, the authors observed an association between IL and BLA and NAc shell with higher doses of morphine. These results suggest the recruitment of different neuronal projections between cortical and accumbal subregions and BLA after different doses of morphine and the balance between these connections may favor drug use vs. avoidance and result in continued use.

Haggerty et al. investigated in mice the impact of prenatal methadone exposure (PME) on the brain and behavioral development of neonates. The study revealed that PME altered the proteome and phosphoproteome of the primary motor cortex (M1) and other connected brain regions, including the primary somatosensory cortex (S1), dorsolateral striatum (DLS), and dorsomedial striatum (DMS). PME was found to affect glutamatergic synapses in M1, with increased glutamate synapse density and decreased glutamate receptor expression. The study suggests that the persistent neuroadaptations in M1 and connected brain regions due to prenatal opioid exposure (POE) may contribute to deficits in motor behavior function throughout the lifespan. Deciphering these changes could lead to novel treatments for mitigating the negative impact of POE on motor and other developmental features.

The orexinergic system is critically involved in reward-related behavior and significantly alters stress responses. Using drug self-administration in rats, Illenberger et al. assessed the effect of an orally administered orexin receptor antagonist, suvorexant (SUV; 0–20 mg/kg) on intravenous escalation of oxycodone self-administration (0.15 mg/kg, 8 h daily) and reinstatement elicited by a contextual/discriminative stimulus in rats. They reported that female rats self-administered oxycodone twice as much as male rats. SUV administration blocked cue-induced reinstatement of oxycodone SA in male and reduced it in female. The highest dose of SUV also reduced oxycodone SA. As the authors indicated, this class of drugs can be repurposed to treat opioid use disorder.

Piekielna-Ciesielska et al. described the synthesis and evaluated thirteen endomorphin 2 (EM-2) analogs with modifications in positions 1, 2, and/or 3, which displayed high affinity toward the mu opioid receptor similar to EM-2 in the calcium mobilization assay. Log *p* values revealed that all of the analogs had somewhat increased lipophilicity and stability compared to EM-2. All experiments were *in vitro* and examined affinity and activity. Using BRET, the authors showed that nearly half of the developed analogs exhibited bias toward G-protein with no preference toward beta-arrestin 2 recruitment. Considering that G-protein biased compounds could possess analgesia with limited side effects, these compounds could provide the best tools to test this possibility.

Despite the recent development in pain management, opioids remain the mainstay for the treatment of moderate to severe pain. To reduce their abuse potential, attempt have been made to develop several classes of novel opioids acting at mu opioid peptide receptors (MOP) and other receptors, called bifunctional compounds have been developed, showing promise as analgesics with reduced tolerance and dependence liability. However, the effects of these novel compounds on respiratory, gastrointestinal, and cardiovascular systems have not been fully characterized. Further studies are needed to determine their impact on the brain reward circuit, as well as to assess the behavioral, neurochemical, and molecular changes following chronic use and their implications in substance use disorders. Additionally, it is essential to explore whether the addition of these novel small molecules would hinder or promote overdose and death associated with opioid use. In light of these knowledge gaps, further investigation of these bifunctional compounds is crucial to unveil their full therapeutic potential and safety profile, paving the way for improved pain management and addiction treatment strategies.

Despite the recent development in the field of opioids, further research is needed to develop safer analgesics with reduced or no abuse liability or adjunct therapeutic agents, such as SUV, that reduce abuse potential of opioids.

